# Predictive Value of Preoperative ICG-R15 Testing in Post-hepatectomy Liver Failure Following Major Liver Resection: Indian Experience

**DOI:** 10.1007/s13193-024-01884-3

**Published:** 2024-01-23

**Authors:** Subha Sampath, Shraddha Patkar, Jasmine Agarwal, Kinjalka Ghosh, Tanuja Shet, Kunal Gala, Nitin Shetty, Mahesh Goel

**Affiliations:** 1 Department of Surgical Oncology, Tata Memorial Hospital, Homi Bhabha National Institute, Parel, Mumbai, Maharashtra India; 2 Division of Hepatobiliary Surgical Oncology, Department of Surgical Oncology, Tata Memorial Hospital, Homi Bhabha National Institute, Parel, Mumbai, Maharashtra India; 3 Department of Biochemistry, Tata Memorial Hospital, Homi Bhabha National Institute, Parel, Mumbai, Maharashtra India; 4 Department of Pathology, Tata Memorial Hospital, Homi Bhabha National Institute, Parel, Mumbai, Maharashtra India; 5 Department of Radiodiagnosis, Tata Memorial Hospital, Homi Bhabha National Institute, Parel, Mumbai, Maharashtra India

**Keywords:** Liver cancer, Post-hepatectomy liver failure, Indocyanine green retention test, Liver function assessment, Risk assessment

## Abstract

Surgical resection stands as the preeminent therapeutic approach for both primary hepatocellular carcinoma and metastatic liver malignancies. Its efficacy is contingent upon the attainment of a comprehensive excision while ensuring a sufficient future liver remnant (FLR). However, post-hepatectomy liver failure (PHLF) remains a significant challenge, particularly in patients with preexisting liver disease. The present study aims to investigate the predictive value of the preoperative indocyanine green retention test at 15 min (ICG-R15) in identifying patients at risk of PHLF following major liver resection. This retrospective review focused on patients who underwent the ICG-R15 test before major liver resection between August 2021 and January 2023. All patients underwent standard preoperative evaluation and staging. Patients with primary or metastatic liver cancer planned for major resection and undergoing ICG-R15 were included in the study. Patients with elevated serum bilirubin (> 3 mg/dl) and those not undergoing liver resection or minor liver resection (< 3 segments) were excluded from the study. PHLF was defined by the International Study Group of Liver Surgery (ISGLS) criteria. Follow-up was performed to identify 90-day morbidity. Using univariate and multivariate logistic regression analyses, we confirmed independent risk parameters that predicted postoperative major complications and severe PHLF. The study included 72 patients who underwent preoperative ICG-R15 testing prior to major liver resection. PHLF occurred in 28 patients (38.9%), with 24 patients (33.3%) classified as severity score B and 3 patients (4.16%) had severity score C. Univariate analysis revealed future liver remnant (FLR), ICG-R15, and blood transfusion as predictors of PHLF. Multivariate analysis confirmed FLR (*p* = 0.019) and ICG-R15 (*p* = 0.032) as significant predictors. Receiver operating characteristic curve analysis yielded an area under the curve of 0.642 for ICG-R15 in predicting PHLF. An optimal cut-point of 7.5 was determined. Our study highlights the importance of preoperative risk assessment of liver function evaluation using the ICG-R15 test, to predict the risk of PHLF following liver resection. Implementing appropriate interventions, especially in patients with borderline FLR, can improve surgical outcomes and enhance patient safety. Further research and prospective studies are essential to refine risk prediction models and improve rates of PHLF after liver resections.

## Introduction

Surgical resection is considered the treatment of choice for primary liver cancer and metastatic liver cancer, provided a R0 resection can be performed with adequate future liver remnant (FLR) [1]. However, post-hepatectomy liver failure (PHLF) poses a formidable challenge with high morbidity and mortality [1]. While uncompromised livers with smaller resections exhibit a correlation between postoperative function and remnant liver function, this relationship does not hold true for patients with preexisting parenchymal disease. The size and quality of the FLR are crucial determinants of postoperative outcomes.

Various methods are available to assess liver function, including biochemical studies, grading systems, volumetric analysis, and scintigraphic studies. Among these, the indocyanine green retention test at 15 min (ICG-R15) has emerged as a quantitative excretory hepatic functional method to evaluate functional hepatocytes and liver blood flow, especially in Asian series of patients with different hepatic diseases [2]. Preoperative ICG-R15 testing has gained significant popularity and validation in East Asian countries, including Japan and South Korea, where it has become an integral part of preoperative evaluation protocols [2–5]. In contrast, the use of ICG-R15 in Western medical practice has been relatively limited, with fewer studies and clinical validations available in the literature. Despite the availability of ICG at affordable cost, Indian data on the use of preoperative ICG-R15 to predict PHLF is scarce. Only one Indian study has reported the use of the ICG clearance test and compared it with the model for end-stage liver disease (MELD) score in patients with liver cirrhosis and found that ICG-R15 had higher sensitivity and specificity than the MELD score for assessing the prognosis of these patients [6]. To the best of our knowledge, this is the first Indian study to comprehensively assess the significance of preoperative ICG-R15 in predicting PHLF in patients undergoing major liver resections. Understanding the predictive value of this parameter can offer valuable insights into improving surgical outcomes and reducing the incidence of PHLF.

## Methods

This is a retrospective analysis of a prospectively maintained database. Electronic medical records of patients who underwent ICG-R15 test prior to major liver resection between August 2021 and January 2023 were reviewed.

Patients planned for liver resection underwent complete pre-operative evaluation with triphasic contrast-enhanced computed tomography (CECT) of the abdomen and pelvis and/or gadolinium-enhanced magnetic resonance imaging (MRI) of the liver along with CT chest. Evaluation of portal hypertension, Child-Turcotte-Pugh scoring, and staging with Barcelona Clinic Liver Cancer (BCLC) and Hong Kong Liver Cancer (HKLC) were additionally done for patients with HCC [7, 8]. After discussion in the multi-disciplinary liver clinic, all potential patients for surgery were subjected to an ICG clearance test.

In this study, we enrolled patients diagnosed with primary liver cancer or metastatic liver disease who were scheduled for major liver resection and subsequently underwent the ICG clearance test. The extent of liver resection was determined by the size, location, and proximity to peripheral vascular structures of the tumor. Major liver resection was defined as resection of 3 or more Couinaud segments [9].

**Fig. 1:**
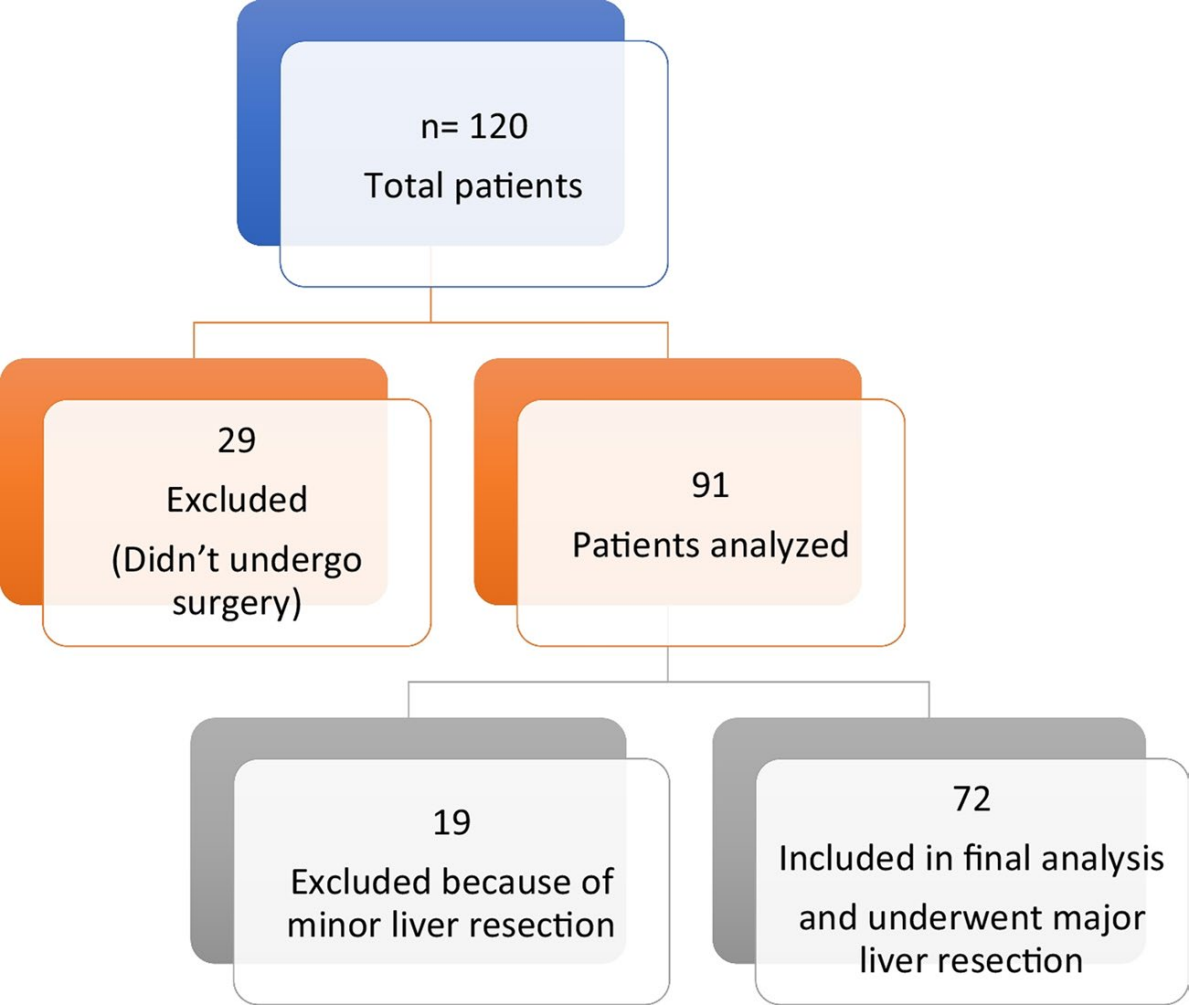
Consort diagram representing study population

Patients with elevated preoperative bilirubin levels (> 3 mg/dl) who could not undergo ICG retention testing, as well as those who did not undergo liver resection due to reasons such as patient default, interim progression, surgical ineligibility, or a change in the treatment plan, were excluded from the study. Those patients who underwent minor liver resections (less than three segments) were also excluded (Fig. 1).

Post-hepatectomy liver failure (PHLF) was defined and graded as per the International Study Group of Liver Surgery (ISGLS) criteria [10]. Patients with hyperbilirubinemia and abnormal coagulation on postoperative day 5 were defined as post-hepatectomy liver failure (PHLF). The patients were followed up for period of 3 months to document 90-day morbidity.

### Protocol for ICG 15 Test

Patients were advised overnight fasting for 12 h prior to day of ICG testing in which two vials (3 ml) of blood sample were collected at the start of the test (labelled as 0 min for blanking), followed by injection of ICG (0.5 mg/kg, Aurogreen Health Private Ltd) through a peripheral vein of forearm. Serial collection of venous blood at 5-min intervals for 20 min was done and ICGR-15 (ICG retention rate at 15 min) was measured by Motras scientific 1600UV spectrophotometer® (registered trademark of Motras Scientific Instruments).

### Statistical Analysis

Categorical variables were shown as frequencies. Continuous variables were shown as median (Q25–Q75). Using univariate and multivariate logistic regression analyses, we confirmed independent risk parameters that predicted postoperative major complications and severe PHLF. For continuous variables, a chi-square test was used, and for categorical variables, a *t*-test was used. A *p* value of < 0.05 was considered significant.

## Results

During the study period, 120 patients planned for major liver resection underwent preoperative ICG-R15 testing. Of these, 29 patients were excluded due to various reasons, mentioned above. Additionally, 19 patients underwent minor liver resection and were excluded, leaving 72 patients for final analysis (Fig. 1). The majority of patients were male (*n* = 50, 69.4%), and the median age at presentation was 53 years (range 22–79 years). Chronic hepatitis was seen in 10 patients (Table 1). Cirrhosis was observed in 16 patients (22.2%), with 13 patients (18.1%) classified as CTPA-5 and 3 patients (4.2%) as CTPA-6. Among the non-cirrhotic patients (*n* = 56), primary liver tumors were the most prevalent (*n* = 29), followed by metastatic liver tumors (*n* = 16), perihilar cholangiocarcinoma (*n* = 6), and benign pathology (*n* = 5).

**Table 1:** Patient demographics

		PHLF-no	PHLF-yes	*p* value
Sex (*N*, %)				
Male	50 (69.4%)	26	24	0.017
Female	22 (30.6%)	18	4	
Median age (years, range)	53 (22–79)	55 (22–79)	58 (32–78)	0.26
Mean weight (kg, range)	59 (39–84)	56 (40–83)	63.5 (39–84)	0.02
History of chronic hepatitis (%)				
No	62 (86.1)	40	22	
Hepatitis B	9 (12.5)	4	5	0.23
Hepatitis C	1 (1.4)	0	1	
Cirrhosis				
Absent	56 (77.8)	37	19	
Present	16 (22.2)	7	9	0.10
CTP class among cirrhotics (*N*, %)				
CTP-A5	13 (81.3)	6	7	0.24
CTP-A6	3 (18.7)	1	2	
ICG-R15 median (range)	7.38 (0.5–18)	6.84 (0.5–15.9)	10.1 (2.02–18)	0.04
Median FLR	53 (30–89)	55 (30–89)	48.5 (30–75)	0.02

Abbreviations; *CTP*, Child-Turcotte-Pugh; *ICG-R15*, indigo cyanine green, retention at 15 min; *PHLF*, post-hepatectomy hepatic failure; *FLR*, future liver remnant

In the cirrhotic patient group (*n* = 16), primary liver tumors were notably predominant (*n* = 13), while fewer cases were observed for metastatic liver tumor (*n* = 1), perihilar cholangiocarcinoma (*n* = 1), and benign pathology (*n* = 1).

The median ICG-R15 was 7.38 (range 0.5–18%). The median ICG-R15 in patients with and without PHLF was 6.84 and 10.1% respectively. Among non-cirrhotic patients developing PHLF (*n* = 19), those who underwent extended resection (*n* = 2) displayed a median ICG-R15 of 7.14, whereas non-cirrhotic patients without extended resection (*n* = 17) exhibited a median ICG-R15 of 9.8. Though there was a discernable difference between the two groups, nevertheless, the interpretation of these findings is challenged by the modest sample sizes and a skewed distribution, making definitive conclusions difficult. Patient demographics are summarized in Table 1

Hepatocellular cancer was the most common indication for surgery. Right hepatectomy was the most frequently performed resection in 41 patients (36.9%). The median operating time was 270 min (range 180–480 min). The median blood loss across all study participants was 2400 ml, with those without post-hepatectomy liver failure (PHLF) experiencing 1800 ml, and patients with PHLF encountering 2500 ml blood loss. The median blood loss in our study may exceed values reported in literature, because, as an institutional policy, we do not routinely practice clamping at the porta during liver resection. This is aimed at mitigating postoperative liver decompensation rates. This may potentially lead to increased blood loss. Intraoperative details are presented in Table 2

**Table 2:** Intraoperative details

Characteristic	Value	PHLF-no	PHLF-yes	*p*-value
Operation time (minutes, range)	330 (180–480)	300 (180–450)	270 (180–480)	0.31
Median intraoperative blood loss (milliliters)	2400 (500–15,000)	1800 (500–15,000)	2500 (800–8000)	0.414
Blood transfusion (per patient)				
Concentrated red blood cells (units, range)	1.9 (0–10)	1.4 (0–10)	2.75 (0–9)	0.01
Type of surgery (*N*, %)		(44)	(28)	
Right hepatectomy	41 (56.9%)	23	18	0.24
Left hepatectomy	25 (34.7%)	19	6	
Extended right hepatectomy	1 (1.38%)	0	1	
Extended left hepatectomy	3 (4.10%)	2	1	
Central hepatectomy	2 (2.70%)	0	2	
Histopathology				
Primary liver tumor	42 (58.4%)	26	16	0.28
pHCC	7 (9.7%)	2	5	
Liver metastasis	17 (23.6%)	12	5	
Benign	6 (8.3%)	4	2	

Abbreviations: *pHCC*, peripheral hepatocellular carcinoma

### Predictive Factors for PHLF

Out of the 72 patients analyzed, 28 patients (38.9%) developed PHLF (Table 3), and among them, 24 patients (85.7%) had a severity score B, and 3 patients (10.7%) had a severity score C (Table 3). The mean ICG-R15 was lower for patients without PHLF (7.17, range 0.50–15.9) compared to patients who developed PHLF (9.79, range 2.02–18).

**Table 3:** PHLF development

PHLF development		(*N*, %)
No		44 (61.1)
Yes	Total	28 (38.9)
	PHLF-A	01 (3.5)
	PHLF-B	24 (85.7)
	PHLF-C	03 (10.7)

Abbreviation: *PHLF*, post-hepatectomy liver failure

Various factors were analyzed for their ability to predict PHLF (Table 4), including age, FLR, ICG-R15, blood transfusion, cirrhosis, and extended resections. On univariate analysis, FLR (*p*, 0.01), ICG-R15 (*p*, 0.02), and blood transfusions (*p*, 0.01) were identified as predictors of PHLF. However, on multivariate analysis, FLR (*p*, 0.019) and ICG-R15 (*p*, 0.032) were confirmed to be significant predictors of PHL.

**Table 4:** Univariate and multivariate analyses to identify factors predicting postoperative major complications

Variables	Univariate logistic regression		Multivariate logistic regression	
	HR (95% CI)	*p* value	HR (95% CI)	*p* value
Age	1.02 (0.98, 1.06)	0.16		
FLR	0.95 (0.91, 0.99)	**0.01**	0.96 (0.91–0.99)	**0.019**
ICG-R15	1.1 (1.02–1.31)	**0.02**	1.1 (1.01–1.34)	**0.032**
Blood transfusions	1.3 (1.0–1.7)	**0.01**		
Cirrhosis	0.39 (0.12–1.2)	0.11	2.9 (0.89–9.3)	0.075
Extended Resections	0.61 (0.08–4.6)	0.64	0.63 (0.07–5.3)	0.67

Abbreviations: *FLR*, future liver remnant; *ICG-R15*, indigo cyanine green, retention at 15 min

Values in bold indicate significant *p* values

### 90-day Morbidity

Among the 72 patients included in the analysis, 28 individuals (38.9%) developed post-hepatectomy liver failure (PHLF), as detailed in Table 3. Within this cohort, 24 patients (85.7%) exhibited severity score B, while 3 patients (10.7%) were classified with severity score C, as previously outlined in the manuscript.

Of the 24 patients with PHLF severity score B, 3 individuals experienced morbidity of Clavien-Dindo grade (1–3) at the 90-day mark. Two of these cases involved persistent PHLF B, necessitating ongoing management with diuretics, while the third case resulted from a biliary leak requiring pigtail insertion. An additional patient, classified with Clavien-Dindo grade >3 at 90 days, had a repeat admission and required intensive care due to hepatic encephalopathy but ultimately achieved successful recovery.

Among the 3 patients with PHLF severity score C, 2 unfortunately succumbed during the postoperative period. One patient with PHLF C exhibited Clavien-Dindo grade >3 due to a persisting biliary fistula which needed re-exploration surgery.

## Discussion

PHLF is a major complication after liver resection and has been reported to occur in 1.2–32% of patients, with corresponding mortality rates of up to 5.0% [11–14]. Predicting PHLF preoperatively is of paramount significance, as identifying patients with a high predicted risk of PHLF can help them either avoid surgery altogether or undergo preoperative procedures to increase the liver reserve, such as portal vein embolization, dual vein embolization, or associating liver partition and portal vein ligation (ALPPS).

The assessment of the liver for surgery encompasses both anatomical and functional evaluations. While accurate methods such as CT volumetry [15] and specialized software like *Myrian*® (registered trademark of Intrasense’s medical imaging review) and Synapse® 3D® (registered trademark of Fujifilm) exist for determining anatomical suitability for resection, the realm of functional assessment still lacks a singular dependable approach, despite remarkable progress in medical technology. In this context, the ICG-R15 test promises to be a straightforward and trustworthy option for evaluating the functional sufficiency of the remaining liver tissue.

Indocyanine green (ICG) is a highly plasma protein-bound, water-soluble anionic organic tricarbocyanine dye. After intravenous injection, it is taken up by the hepatocytes and subsequently removed from the blood exclusively by the liver, excreted into the bile without intrahepatic biotransformation [16]. The ICG retention test at 15 min (ICG-R15) has emerged as a reliable method to assess functional hepatocytes and liver blood flow. It is a quantitative excretory hepatic functional test that evaluates the liver’s capacity to regenerate and predicts function after resection [2–4]. Compared to other dynamic techniques, the ICG-R15 test is more reproducible, and non-invasive, making it valuable. However, there are few reports on the ICG-R15 test in predicting the incidence of PHLF in patients operated for hilar cholangiocarcinoma [17] and for patients of HBV HCC [18].

In the present study, approximately 38% of patients who underwent liver resection developed PHLF with a clinically relevant PHLF rate of 33.33% (severity score B and C), which is consistent with the incidence reported by others [19, 20]. In univariate analysis, intraoperative blood transfusion was a significant factor in predicting PHLF (*p*-value, 0.01); however, it lost its significance in multivariate analysis. This finding is contrary to many other studies [19, 21, 22] that reported an increased risk of PHLF after blood transfusions.

Studies have established a strong correlation between FLR and the occurrence of PHLF [23]. Patients with a low FLR are at heightened risk of developing PHLF after liver surgery, as the residual liver tissue may be unable to compensate for the loss of the resected portion [24]. Identifying patients with insufficient FLR and intervening with strategies like portal vein embolization or ALPPS can enhance FLR and minimize the risk of PHLF [25]. Our results align with these findings, as FLR showed a strong correlation with PHLF in both univariate and multivariate analyses.

**Fig. 2:**
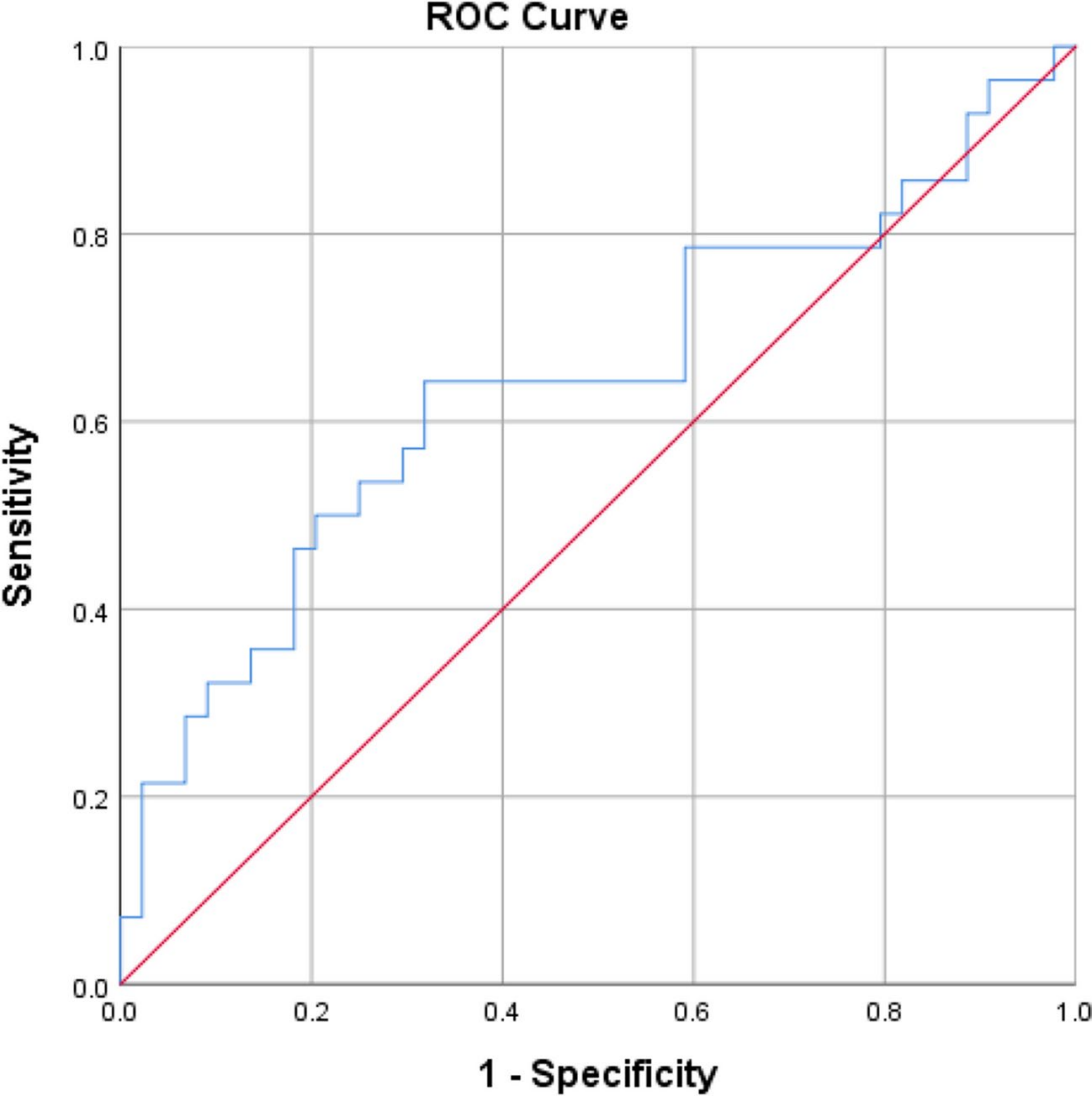
ROC curve for ICG-R15

In the present study, the median ICG-R15 was lower for patients without PHLF (6.84, range 0.50–15.9) compared to patients who developed PHLF (10.1, range 2.02–18). This finding is consistent with previous studies [4]. The weighted mean ICG-R15 under the random effects model was 11 in the meta-analysis by Granieri et al. [26]. The area under the ROC curve (AUC) (Table 5, Fig. 2) was 0.642 (95% CI, 0.503–0.781), favoring the use of ICG-R15 to predict PHLF. Granieri and colleagues in their meta-analysis [26] summarized papers reporting AUC values ranging from 0.630 to 0.730, with a pooled AUC of 0.673 (95% CI: 0.632–0.713). The lower AUC could be attributed to heterogeneity among the studies with some reporting high and others reporting low AUC values.

**Table 5:** Area under curve

Test result variable(s): ICG_R15		
Area	Std. error^a^	Asymptotic Sig.^b^
.642	.071	.043

^a^Under the nonparametric assumption

^b^Null hypothesis: true area = 0.5

The optimal cut-point value determined by ROC analysis in our study was 7.5. Patients above this threshold have a higher risk of developing PHLF (*p* = 0.03) (Table 6). This value is slightly higher than that reported by Schwarz and colleagues [5], with a cut-point of 5.6%.

**Table 6:** Co-relation between optimal ICG-R15 value and development of PHLF

		ICG_category		Total
		< 7.5	> 7.5	
PHLF	No	27	17	44
	Yes	10	18	28
Total		37	35	72
				*p* value − 0.03

Abbreviations: *PHLF*, post-hepatectomy liver failure

A wide variety of factors affects the test including intrahepatic shunting, sinusoidal capillarization, and hyperbilirubinemia. Regarding the latter, it is worth noting that bilirubin and ICG compete for the same plasmatic transporter that results in a reduced ICG uptake in patients suffering from obstructive jaundice (26).

We acknowledge some limitations in this study. This was a retrospective observational study with inevitable biases for patient selection. Additionally, the study was limited to the immediate postoperative period and does not report on long-term outcomes. The study has a low ROC of 0.642 with sensitivity and specificity of 70% which may impede accuracy. Going by the ROC figure, a sensitivity and specificity of 70% have been achieved with an area under a curve of 0.642 which is satisfactory for this as a single investigation. The limited sample size, diverse range of diagnoses (including some with “n” less than 10), and a substantial proportion of patients with normal liver conditions may impede the ability to draw definitive conclusions.

## Conclusion

Our study highlights the importance of preoperative risk assessment of liver function evaluation using the ICG-R15 test, to predict the risk of PHLF following liver resection. Implementing appropriate interventions, especially in patients with borderline FLR, can improve surgical outcomes and enhance patient safety. Further research and prospective studies are essential to refine risk prediction models and improve rates of PHLF after liver resections.
